# The Maghreb – one more important biodiversity hot spot for tiger beetle fauna (Coleoptera, Carabidae, Cicindelinae) in the Mediterranean region

**DOI:** 10.3897/zookeys.482.8831

**Published:** 2015-02-12

**Authors:** Radomir Jaskuła

**Affiliations:** 1 Department of Invertebrate Zoology & Hydrobiology, University of Łódź, Banacha 12/16, 90-237 Łódź, Poland

**Keywords:** Coleoptera, Cicindelinae, tiger beetles, zoogeography, biodiversity, species richness, habitat preferences, Mediterranean region, North Africa, Morocco, Algeria, Tunisia

## Abstract

The tiger beetle fauna of the Maghreb region is one of the richest in the Palaearctic, including 22 species and 5 subspecies and 19% of all Palaearctic species of Cicindelinae. Assembled to their chorotypes, the Maghreb tiger beetles fall into eight different groups that include Maghreb endemics (26% of fauna), Mediterranean (7%), West Mediterranean (40%), North African (4%), Mediterranean-Westturanian (4%), West Palaearctic (4%), Afrotropico-Indo-Mediterranean (4%), and Saharian (11%) species. The Mediterranean Sclerophyl and Atlas Steppe are the Maghreb biogeographical provinces with the highest species richness, while the Sahara Desert has the lowest Cicindelinae diversity. Twenty-five cicindelid species and subspecies (93% of Maghreb fauna) are restricted to only one or two habitat types in lowland areas. Only *Calomera littoralis littoralis* and *Lophyra flexuosa flexuosa* are recognized as eurytopic species and occur in three types of habitat. The highest tiger beetle diversity characterizes salt marshes and river banks (in both cases 11 species and subspecies or 41% of Maghreb fauna). Approximately 85% of all Maghreb tiger beetle species and subspecies are found in habitats potentially endangered by human activity.

## Introduction

Tiger beetles (Carabidae: Cicindelinae) include approximately 2600 species of small to medium-sized beetles (Pearson and Cassola 2005). They are predatory with word-wide distributions, excepting some oof the ceanic islands and polar regions (Cassola and Pearson 2000; Pearson and Vogler 2001). Most prefer various sandy habitats where both larvae and adult beetles live. Many recent studies from different continents show that most cicindelid taxa have very narrow habitat specialization and can be found only in one or at most in few very similar types of macrohabitats. As a consequence, tiger beetles have become a very important global flagship group for beetle and insect conservation, often used as biological indicators for determining both regional and global patterns of biodiversity (Knisley and Hill 1992; Pearson and Cassola 1992, 2005; Kitching 1996; Carroll and Pearson 1998a, 1998b; Andriamampianina et al. 2000; Pearson and Vogler 2001; Arndt et al. 2005; Jaskuła 2011). Moreover, as both adults and larvae of cicindelid beetles are predators that prey on different small invertebrates, they can be used for biological control of pests causing important economic destruction (Rodriquez et al. 1988).

The Maghreb is a part of the Mediterranean region, which is known as one of the 25 most important word biodiversity hot spots (Myers et al. 2000; Cuttelod et al. 2008). Concluding from the recent studies upon different plant and animal groups it is also a very important terrestrial Pleistocene glacial refugium, both on the local scale (Husemann et al. 2014) and for the whole Western Palaearctic (Hewitt 1996, 1999; Thomson 2005; Blondel et al. 2010; Habel et al. 2010). High levels of biodiversity in the Maghreb region can be explained by the mosaic heterogeneous landscapes occurring in this area as well as by relatively high climatic stabilization of this region (Blondel et al. 2010). Moreover, Maghreb served as an important natural bridge for historical and present dispersal between Africa and Europe, mainly via the Gibraltar and Sicily sea straits which are known as important biogeographical links between both continents at different times (Habel et al. 2010).

The first data on tiger beetle fauna of the Maghreb region were published in the second half of 18^th^ and at the beginning of 19^th^ centuries (e.g. Linnaeus 1758; Fabricius 1781, 1787, 1801; Vigors 1825; Dejean 1831). Since then, more than 80 papers have been published on this topic. Unfortunately, in most cases they include only single faunistic records or data on a single species. Till recently the knowledge on diversity and distribution of tiger beetle fauna was summarized for Tunisia by Korell and Cassola (1987) and Jaskuła and Rewicz (in prep.) and for Morocco by Cassola (1973) and Jaskuła et al. (in prep.).

The paper is the second part of wider studies concerning biodiversity and zoogeography of tiger beetle fauna of the Mediterranean region (Jaskuła 2011). Its aim is to summarize the knowledge on diversity of tiger beetles in the Maghreb region with particular emphasis on the group diversity, distribution, zoogeographical composition as well as the macrohabitat preferences of cicindelid taxa.

## Study area

Here, the Maghreb region is defined as a part of northwestern Africa with its northern boundary made up of the Mediterranean Sea, the western boundary at Atlantic coast, the southern at Sahara Desert, and the eastern at the political border of Tunisia and Libya (Michard et al. 2008, Fig. 1). Politically the area includes four countries, three of them completely confined to the Maghreb region (Tunisia, Algeria, and Morocco) as well as the two small enclaves (Ceuta and Melilla) belonging to Spain. The region has a total area of 2,991,933 km^2^, which is nearly 10% of the entire African continent and some 5.5% of the Palaearctic ecozone.

**Figure 1.**
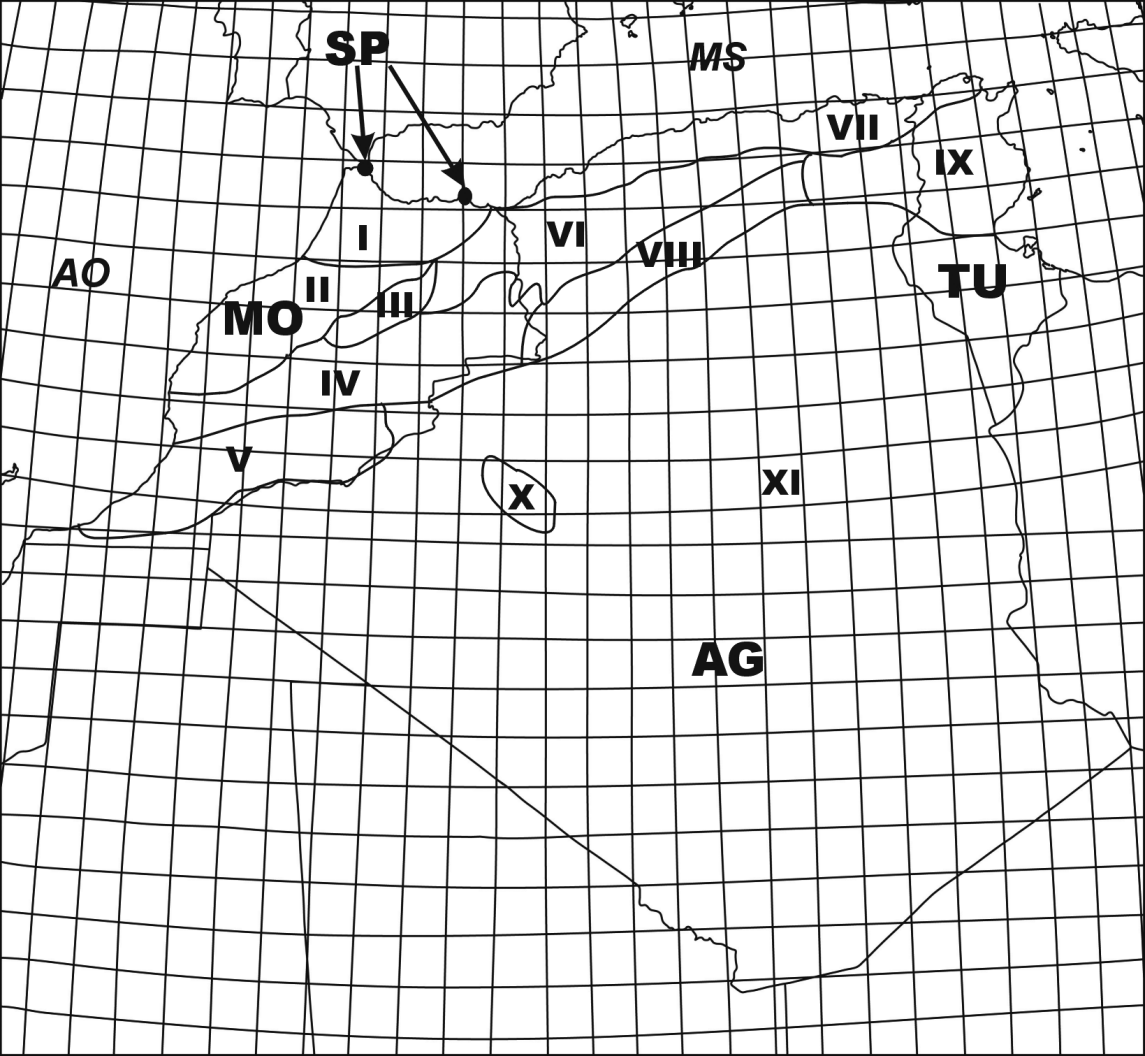
Geographical and administrative divisions of the Maghreb region: I – Rif, II – Central Massif, III – Middle Atlas, IV – High Atlas, V – Anti-Atlas, VI – High Plateaus, VII – Tell Kabyllas, VIII – Saharan Atlas, IX – Tunisian Atlas, X – Ougarta, XI – Saharan Platform, MO – Morocco, AG – Algeria, TU – Tunisia, SP – Spain (Ceuta and Melilla), AO – Atlantic Ocean, MS – Mediterranean Sea.

The largest surface of the Maghreb region is montane. Lowlands extend only along the lower reaches of rivers that are grouped into two drainages of the Mediterranean Sea and of the Atlantic Ocean (Woodward 2009). Geographically the Maghreb is divided into the following main regions: Rif, Central Massif, Anti-Atlas, Middle Atlas, Tell Kabyllas, High Plateaus, Tunisian Atlas, Saharan Atlas, High Atlas, Ougarta, and Saharan Platform (Michard et al. 2008; Fig. 1).

**Figure 2.**
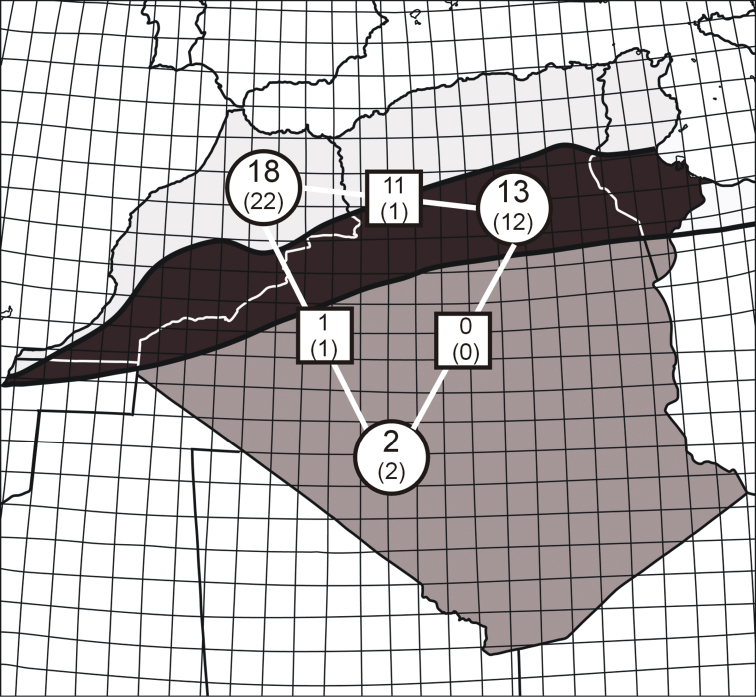
Tiger beetle faunas in the biogeographical provinces of the Maghreb region (division after Udvardy 1975): light grey – Mediterranean Sclerophyl, black – Atlas Steppe, dark grey – Sahara Desert. Numbers in the circles indicate the number of cicindelid taxa for the separate regions and the squares give the number of taxa common to the provinces shared (large – species, small in brackets – subspecies).

According to biogeographical divisions by Udvardy (1975) the Maghreb region belongs to three provinces (Fig. 2): Mediterranean Sclerophyl – which includes Mediterranean coasts of Tunisia, Algeria, and Morocco as well as entire areas of Spanish enclaves, Ceuta and Melilla; the Atlas Steppe – with the highest montane areas of Tunisia, Algeria, and Morocco; and the Sahara Desert – the biggest area of Maghreb with the southernmost parts of Algeria and Tunisia.

## Material and methods

The basis for the analysis of tiger beetle fauna of the Maghreb region comes from published literature data, museum collections (Museum and Institute of Zoology, Polish Academy of Science, Warsaw, Poland; Museum of Natural History, Vienna, Austria; Royal Belgium Institute of Natural Sciences, Brussels, Belgium), and the author's collections gathered during two scientific trips covering almost entire areas of Morocco and Tunisia (2^nd^ and 4^th^ TB-Quest Expeditions) in 2009 and 2010. Additionally, single specimens were studied thanks to Dr. J. Ch. Habel (Germany). Recently all these faunistic data were summarized in two papers on tiger beetle species of Tunisia and Morocco (Jaskuła and Rewicz in prep., Jaskuła et al. in prep.).

All the statistical analyses used in this paper follow my previous work on tiger beetles occurring in the Mediterranean region (Jaskuła 2011) to enable proper comparison. The tiger beetle species richness and distribution of taxa were analyzed based on squares of 1° longitude and latitude. In each square the total number of all species recorded was summarized. Similarities among tiger beetle fauna between geographical divisions of the Maghreb region were measured using the Bray-Curtis index for presence/absence data (Primer v. 5.0) as well as Jaccard’s (1902) index was used to present the degree of dissimilarity between zoogeographic regions proposed by Udvardy (1975):

R = 100c/a+b-c

where: a – number of species in the richest fauna, b – number of species in the poorest fauna, c – number of species common to both faunas.

Chorotype definition follows Vigna Taglianti et al. (1999).

## Results

### Diversity of tiger beetles in Maghreb region

According to Werner (1991, 1992), Putchkov and Matalin (2003), Lopéz et al. (2006), Cassola and Wiesner (2009), and Deuve (2011, 2012) approximately 110 tiger beetle species occur in Palaearctic (species recorded by Putchkov and Matalin (2003) in the oriental part of China and in Taiwan were excluded). Out of that, 22 species and 5 subspecies have been found in the area of Maghreb region (Table 1) which is approximately 19% of all the Palaearctic tiger beetle species.

**Table 1.** Chorotypes of Maghreb tiger beetles (based on Vigna Taglianti et al. 1999).

Region	Species
Maghreb endemics	*Platydela coquerelii coquerelii*, *Platydela coquerelii theryi*, *Platydela segonzaci*, *Neolaphyra leucosticta leucosticta*, *Neolaphyra leucosticta simulans*, *Neolaphyra peletieri*, *Neolaphya truquii*
West Mediterranean	*Calomera littoralis littoralis*, *Calomera lunulata*, *Cassolaia maura maura*, *Cassolaia maura cupreothoracica*, *Cephalota circumdata imperialis*, *Cephalota littorea goudotii*, *Cephalota luctuosa*, *Cicindela campestris atlantis*, *Cicindela maroccana maroccana*, *Cylindera trisignata trisignata*, *Cylindera trisignata siciliensis*
North African	*Cephalota tibialis lyonii*
Mediterranean	*Calomera aulica aulica*, *Lophyra flexuosa flexuosa*
Mediterranean-Westturanian	*Grammognatha euphratica euphratica*
West Palaearctic	*Cicindela campestris campestris*
Afrotropico Indo-Mediterranean	*Myriochila melancholica melancholica*
Saharian	*Habrodera leucoptera leucoptera*, *Myriochila dorsata*, *Myriochila mirei*

The Maghreb cicindelid species belong to eleven genera (92% of the Mediterranean or 61% of the Palaearctic fauna) including: *Grammognatha* (1 species, 100% of both Mediterranean and Palaearctic), *Myriochila* (3 species, 75% of Mediterranean and 27% of the Palaearctic), *Habrodera* (1 species, 50% of both Mediterranean and Palaearctic), *Calomera* (3 species, 50% of Mediterranean and 19% of Palaearctic), *Lophyra* (1 species, 50% Mediterranean and 8% of Palaearctic), *Cephalota* (4 species, 40% Mediterranean of and 21% of Palaearctic), *Cassolaia* (1 species, 50% of both Mediterranean and Palaearctic), *Neolaphyra* (3 species, 75% of both Mediterranean and Palaearctic), *Platydela* (2 species, 100% of both Mediterranean and Palaearctic), *Cylindera* (1 species, 8% of Mediterranean and 3% of Palaearctic), and *Cicindela* (1 species, 17% of Mediterranean and 2% of Palaearctic). Comparing to the total list of tiger beetle genera occurring in the Mediterranean region, only the genus *Homodela* (distributed in Syria and the southern part of Turkey) is not present in Maghreb.

Seven tiger beetle taxa belonging to five species (*Platydela coquerelii coquerelii*, *Platydela coquerelii theryi*, *Platydela segonzaci*, *Neolaphyra leucosticta leucosticta*, *Neolaphyra leucosticta simulans*, *Neolaphyra peletieri*, and *Neolaphya truquii*) are endemic to the Maghreb region. Additionally, for three taxa (*Habrodera leucoptera leucoptera*, *Myriochila dorsata*, and *Myriochila mirei*) Maghreb is the only place of occurrence in the Palaearctic ecozone (distributed also south of Sahara) and for four others, this area is the only one in the African part of the Palaearctic (they are known from south-western Europe and/or from south-western Asia).

Based on the chorotypes, tiger beetles of the Maghreb region can be included into eight different groups (Vigna Taglianti et al. 1999; Table 1). Except Maghreb endemics, which constitute 26% of all tiger beetle taxa (species and subspecies) noted from this area, representatives of West Mediterranean (40%), North-African (4%), Mediterranean (7%), Mediterranean-Westturanian (4%), West Palaearctic (4%), Afrotropico Indo-Mediterranean (4%), and Saharian (11%) taxa can be found in this region.

The number of Maghreb tiger beetle species is high if compared with the number noted from other regions of the West Palaearctic with other areas of similar size (Table 2), especially when the large part of Algerian Sahara is excluded (a great part of the Sahara desert is so dry that there are no habitats which would be attractive for Cicindelinae). In this case, the diversity and species richness of the Maghreb tiger beetle fauna is similar not only to the faunas of all the other most important glacial refugia in Europe (Iberian, Balkan, and Italian Peninsula) and in south-western Asia (Turkey), but even to the fauna known from the entire territory of the European part of Russia.

**Table 2.** Comparison of area and tiger beetle species richness of Maghreb and some other regions from Western Palaearctic (data compiled from different sources).

Region	Area (km^2^)	Number of species
Maghreb	2 991 933	22
Maghreb (excluding biogeographical province – Sahara Desert)	ca. 714 500	21
Iberian Peninsula	580 000	19
Balkan Peninsula	550 000	19
Italian Peninsula	150 000	13
France (mainland)	675 000	14
Scandinavian Peninsula	800 000	5
Ukraine	603 700	19
Turkey	783 562	26
Russia (European part)	4 268 850	28

### Distribution of tiger beetles in the Maghreb region

Records from the literature and from my own observations within squares of 1° latitude and longitude show that the species richness of particular regions within the Maghreb differs both in species composition and in number of taxa. The highest number of tiger beetle taxa is found along the sea coasts of the Mediterranean Sea and of the Atlantic Ocean, both according to the geographical divisions and biogeographic regions defined by Udvardy (1975, Figs 2–3). The greatest Cicindelinae species richness in the Maghreb can be found in two biogeographic provinces which are similar in their surface area: the Mediterranean Sclerophyl (18 species or 22 species and subspecies, 82% of the Maghreb fauna) and the Atlas Steppe (13 species or 15 species and subspecies, 56% of fauna). The lowest species richness characterizes the Sahara Desert, where only two species (7% of fauna) have been noted, despite the fact that the Sahara Desert covers a part of Maghreb that is larger than both previous provinces combined.

**Figure 3.**
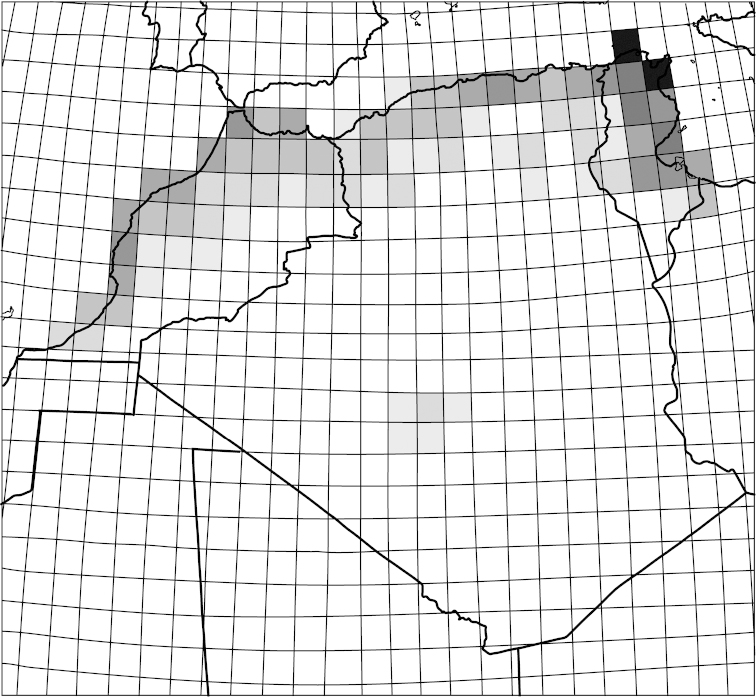
Species richness of tiger beetles within the Maghreb region. The colour gradient indicates an enhanced diversity from zero species (white square) to seven (black square).

**Figure 4.**
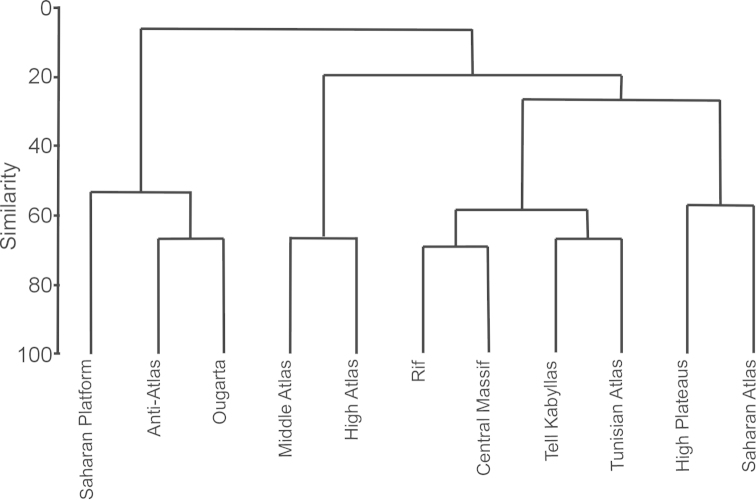
Similarities among tiger beetle faunas inhabiting regions of the Maghreb (Bray-Curtis similarity index for presence/absence data).

The Bray-Curtis analysis of similarities among the tiger beetle faunas from different Maghreb geographical regions shows the presence of four main groups (Fig. 4). One of them includes the Saharan Platform, Anti-Atlas, and Ougarta regions belonging to the southernmost part of Maghreb and covering a great part of Udvardy’s (1975) Sahara Desert and a small part of the Atlas Steppe. The second group composes of the Middle Atlas and the High Atlas regions which include the highest mountains within Maghreb, mentioned in biogeographic studies as the western part of the Atlas Steppe. The third group includes four regions located mainly in lowlands and highlands along the Mediterranean and Atlantic coasts: Rif, Central Massif, Tell Kabyllas and Tunisian Atlas. All these geographical regions belong to Udvardy’s (1975) Mediterranean Sclerophyl. The last region is composed of the High Plateaus and of the Saharan Atlas (both being part of the central part of the Atlas Steppe) and covers the greater part of mountain areas in northern Algeria. The Jaccard’s similarity index for Mediterranean Sclerophyl – Atlas Steppe was 42%, for Mediterranean Sclerophyl – Sahara Desert was 4%, and for Atlas Steppe – Sahara Desert was null.

### Ecotypes of Maghreb tiger beetles

In the tiger beetle fauna of Maghreb the most eurytopic taxa are *Calomera littoralis littoralis* and *Lophyra flexuosa flexuosa* (Table 3), both of them occurring in three types of macrohabitats. Five other species/subspecies have been found to occupy two types of habitats, including *Calomera aulica aulica*, *Cassolaia maura maura*, *Cicindela campestris atlantis*, *Cylindera trisignata trisignata*, and *Cylindera trisignata siciliensis*. Twenty other taxa (74% of Maghreb fauna) are known only from one type of habitat, including *Habrodera leucoptera leucoptera*, *Grammognatha euphratica euphratica*, *Myriochila myriochila myriochila* and all four *Cephalota* species from salt marshes, seven taxa restricted to river banks (*Cicindela maroccana maroccana*, *Myriochila dorsata*, *Myriochila mirei*, and all *Neolaphyra*), four found exclusively in mountain and highland pastures and meadows (*Cicindela campestris campestris*, and all *Platydela*), and one taxon known only from sandy sea beaches (*Calomera aulica aulica*). Among all these tiger beetles, 23 species and subspecies (85%) can be classified as coastal and riverine taxa, occupying habitats close to water, such as salt marshes, banks of rivers and lakes, and sea coasts, while four other taxa (19%) are typical mountain beetles occurring in sandy areas in meadows and pastures at higher elevations.

**Table 3.** Tiger beetles of the Maghreb region and their ecological distribution: 1 – salt marshes, 2 – sandy sea beaches, 3 – sandy-rocky sea beaches, 4 – banks of rivers, 5 – banks of lakes, 6 – oases, 7 – mountain and highland pastures and meadows.

No.	Taxon	Macrohabitat type						
		1	2	3	4	5	6	7
1.	*Calomera aulica aulica* (Dejean, 1831)		+	+				
2.	*Calomera littoralis littoralis* (Fabricius, 1787)	+	+		+			
3.	*Calomera lunulata* (Fabricius, 1781)		+					
4.	*Cassolaia maura cupreothoracica* Korell & Cassola, 1987					+		
5.	*Cassolaia maura maura* (Linnaeus, 1758)				+	+		
6.	*Cephalota circumdata imperialis* (Klug, 1834)	+						
7.	*Cephalota littorea goudotii* (Dejean, 1829)	+						
8.	*Cephalota luctuosa* (Dejean, 1831)	+						
9.	*Cephalota tibialis lyonii* (Vigors, 1825)	+						
10.	*Cicindela campestris atlantis* Mandl, 1944				+			+
11.	*Cicindela campestris campestris* Linnaeus, 1758							+
12.	*Cicindela maroccana maroccana* Fabricius, 1801				+			
13.	*Cylindera trisignata trisignata* (Dejean, 1822)	+	+					
14.	*Cylindera trisignata siciliensis* (W. Horn, 1891)	+	+					
15.	*Grammognatha euphratica euphratica* Latreille & Dejean, 1822	+						
16.	*Habrodera leucoptera leucoptera* (Dejean, 1831)	+						
17.	*Lophyra flexuosa flexuosa* (Fabricius, 1787)	+			+		+	
18.	*Myriochila dorsata* (Brullé, 1834)				+			
19.	*Myriochila melancholica melancholica* (Fabricius, 1798)	+						
20.	*Myriochila mirei* Rivalier, 1961				+			
21.	*Neolaphyra leucosticta leucosticta* (Fairmaire, 1859)				+			
22.	*Neolaphyra leucosticta simulans* (Bedel, 1895)				+			
23.	*Neolaphyra peletieri* (Lucas, 1848)				+			
24.	*Neolaphya truquii* (Guérin-Méneville, 1855)				+			
25.	*Platydela coquerelii coquerelii* (Fairmaire, 1867)							+
26.	*Platydela coquerelii theryi* (Alluad, 1930)							+
27.	*Platydela segonzaci* (Bedel, 1903)							+
Total		11	5	1	11	2	1	5

## Discussion and conclusions

### Diversity and distribution of tiger beetles in the Maghreb region

Compared to the surface area of other regions of the Palaearctic, the diversity of tiger beetles of the Maghreb region is high and constitutes about 19% of all Cicindelinae species known from this biogeographic realm (Putchkov and Matalin 2003; Lopéz et al. 2006; Cassola and Wiesner 2009; Deuve 2011, 2012). This clearly proves an important role of Maghreb as diversity hot spot for tiger beetles, noted earlier for many other different taxonomic groups, including plants, terrestrial and freshwater invertebrates, and vertebrates (eg. Schleich et al. 1996; Beauchard et al. 2003; Omodeo et al. 2003; Thompson 2005; Delforge 2006; Blondel and Médail 2009; Blondel et al. 2010). The high diversity of tiger beetle fauna in this area can be explained by two main factors. First is the topographic position of Maghreb within the West Palaearctic realm as the area was (and still is, mainly because of Gibraltar and Sicily sea straits) an important natural bridge between European and African faunas (eg. Harris et al. 2002; Paulo et al. 2002; Carranza et al. 2004, 2006; Veith et al. 2004; Fritz et al. 2006; Weingartner et al. 2006; Recuero et al. 2007; Habel et al. 2008, 2010; Skog et al. 2009). As a result of these biogeographical links between both continents at different times, presently Maghreb region is inhabited by a mixed tiger beetle fauna with representatives of 22 species belonging to eight different groups according to their chorotypes (Table 1). Some of these taxa presently occur both in Northern Africa and in southern Europe (eg. all Maghreb species of *Calomera*, *Cephalota*, *Cylindera*, and *Grammognatha*) (Putchkov and Matalin 2003; Serrano 2013). Similar patterns in faunal elements have been noted also among other groups of invertebrates, including some groups of insects (Weingartner et al. 2006; Riservato et al. 2009; Habel et al. 2008), scorpions (Gantenbein and Largiadèr 2003), amphibians and reptiles (Busack 1986; Schleich et al. 1996; Alvarez et al. 2000; Cox et al. 2006), mammals (Dobson 1998; Cosson et al. 2005; Temple and Cuttelod 2008; Skog et al. 2009) and plants (Thompson 2005).

The second reason of high level species richness of Maghreb tiger beetle fauna is the high diversity of habitats preferred by this beetle group, including sandy sea beaches, salt marshes, river banks, as well as oases and sandy areas in the mountains.

A relatively high level of landscape mosaic and heterogeneity may also explain the general distribution patters of tiger beetle species within the Maghreb region with higher diversity and species richness in the lowlands. The reason is that sandy habitats preferred both by larvae and adult cicindelid beetles are much more diverse at the sea coasts than in the montane areas. This patterns appears to be typical for this beetle group and it is very similar in other regions of the Mediterranean area (Cassola 1970, 1973; Lisa 2002; Jaskuła 2011; Jaskuła and Rewicz 2014) and also in other regions of the world, including western and northern Australia, and the Indian subcontinent (Pearson and Cassola 1992). In contrast, at higher elevations, the percentage of habitat opportunists can be much higher, as was shown by Bhargav et al. (2008) in studies on tiger beetles of Shivalik in Himachal Pradesh in north western India. In these studies, habitat specialists were found only in few of the studied habitats. Probably it can be explained by much higher homogeneity of that landscape when compared with lowland areas.

### Ecological preferences in Maghreb tiger beetles

The analysis of macrohabitat preferences of Maghreb tiger beetles show that most species have very narrow habitat specialization and occur only in one or at most in two very similar types of habitat. Only two of all the 27 taxa known from this region occupy three different habitats – *Calomera littoralis littoralis* and *Lophyra flexuosa flexuosa* (Table 3). Similar observations were made also in other areas of the Mediterranean region, as well as in some other regions of the world. For example, of 19 tiger beetle species and subspecies noted in the Balkans, only two – *Calomera littoralis nemoralis* and *Calomera aulica aulica* – were recorded respectively from four and three different habitat types (Jaskuła 2011). In Australia, among 29 studied species only two (*Myriochila mastersi* and *Myriochila semicincta*) were found as occurring in several habitat types (Freitag 1979). In the Sulphur Springs Valley (Arizona, USA) only *Cicindelidia nigrocoerulea*, one of 20 species noted during studies, was recorded from more than one habitat type (Knisley and Pearson 1984) and in the Colfax County (New Mexico, USA) only four of 19 species (*Cicindela fulgida*, *Cicindela tranquebarica*, *Cicindelidia punctulata*, and *Cicindelidia nigrocoerulea*,) were noted as habitat generalists occurring in seven different macrohabitat types (Knisley 1984). Similar results were provided also by Acciavatti and Pearson (1989) from the Indian subcontinent where among 151 tiger beetle species only *Calochroa flavomaculata* was noted from several different habitat types. Pearson (1984) noted *Odontocheila annulicornis* as the only one cicindelid taxon (of 29 species) inhabiting more than one forest habitat type in the Tambopata Reserve Zone in Peru. Also data from Japan by Satoh et al. (2006) show that usually only single tiger beetle species are eurytopic. In their studies on riparian Cicindelinae in the Tedori River System only *Cicindela transbaicalica* was distributed widely along the river while two other taxa were restricted to only one habitat type. Moreover, the habitat specialization can be so narrow that species occurrence can be restricted to only a small part of a particular habitat. Sometimes, a different type of habitat/microhabitat is occupied by adult beetles and by the larvae. Ganeshaiah and Belavadi (1986), during their studies of four Asian riverine Cicindelinae species, showed that tiger beetles were segregated distinctly along the river beds according to separate habitats. Similar observations were made also in the USA by Schultz and Hadley (1987) who noted that *Cicindela tranquebarica* preffered dry areas while *Cicindela oregona* occupied mainly stream edges, and by Jaskuła (2011) who observed in the different parts of the Balkans that *Calomera littoralis nemoralis* preferred mainly wet sand on edges of water reservoirs while the drier salt marsh substrate was inhabited by *Cephalota chiloleuca*, *Cephalota chiloleuca circumdata* and *Cylindera trisignata hellenica*. Interesting results were also provided by Satoh and Hori (2005) who found spatial segregation during the larval stage of six Japanese tiger beetles. Each of the studied taxa preferred specific type of microhabitat. Moreover, in most of the species the habitat type was different for larvae and for adult beetles.

Many authors explain such narrow cicindelid specialization to habitat/microhabitat type by morphological (Pearson and Murry 1979; Schultz and Hadley 1987; Satoh et al. 2003; Satoh and Hori 2005; Dangalle et al. 2013), physiological (Schultz and Hadley 1987; Hadley et al. 1990), or behavioural (Knisley and Pearson 1981; Pearson and Lederhouse 1987) adaptations of adults and larvae. Moreover, at least in some cases, the opportunistic feeding behaviour can play an important role in colonization of different habitat types by some eurytopic tiger beetle species. A good example comes from the Balkan Peninsula, where a species previously known as typical predatory beetle and habitat generalist, *Calomera littoralis nemoralis*, was observed on sandy sea beach feeding on plant material (Jaskuła 2013). As the same species (but another subspecies – *Calomera littoralis littoralis*) is also one of the only two habitat generalists known from the Maghreb region, it cannot be excluded that similar feeding behaviour may occur also in the North African population of this species.

Underwood et al. (2009a, 2009b) noted that Mediterranean type of habitats are among the rarest globally and are restricted to only 2% of the Earth's land surface. Most of these areas are endangered by human activity. Within the Mediterranean region, including Maghreb, less than 1% of the land surface is legally protected. Moreover, presently almost all habitat types occupied by the Maghreb tiger beetles (eg. salt marshes, sandy sea beaches, and banks of freshwater reservoirs) are significantly altered and are recognised as globally threatened (eg. Silliman et al. 2009). According to the ecological distribution of Maghreb Cicindelinae (Table 3), at least 85% of the recorded taxa occur in these threatened environments and, as a result, are potentially threatened. Additionally, three other taxa (all belonging to *Platydela*) have very restricted world distributions as they are endemics occurring only in small parts of the Moroccan Atlas mountains. All the above, as well as the fact that the Maghreb is a very important transition zone between Africa and Western Europe where faunal elements of various origin meet, clearly prove the unique character of this region as an important biodiversity hot spot for tiger beetle fauna both in the Mediterranean region and in the Western Palaearctic.
